# Barriers and facilitators to behavioral health follow-up in children screened and referred for Adverse Childhood Experiences (ACEs)

**DOI:** 10.1186/s12913-025-13928-7

**Published:** 2026-01-03

**Authors:** Iesha L. Ticknor, Mercie J. Digangi, Laurel D. Sarfan, Sonya Negriff

**Affiliations:** 1 Kaiser Permanente Bernard J. Tyson School of Medicine, 98 S Los Robles Ave, Pasadena, CA 91101 USA; 2 Southern California Permanente Medical Group, 9353 Imperial , Hwy. Downey, CA 90242 USA; 3 Department of Research & Evaluation, Kaiser Permanente Southern California, 100 S Los Robles Ave, Pasadena, CA 91101 USA

**Keywords:** Adverse childhood experiences, ACEs, Pediatrics, Social work

## Abstract

**Abstract:**

We conducted 20 semi-structured phone interviews with caregivers of children referred to behavioral health after positive ACEs screening. We analyzed the transcripts using inductive coding and thematic analysis.

**Results:**

Three themes regarding barriers to completing behavioral health referrals after ACEs screening were identified: (1) difficulty communicating with behavioral health and navigating the healthcare system, (2) appointment availability and access, and (3) medication concerns. Additionally, four themes regarding facilitators were identified: (1) healthcare system support, (2) family and friend support, (3) previous positive experience with behavioral health, (4) child or caregiver requesting therapy.

**Conclusions:**

Most caregivers reported positive views of the referral process. However, the identified barriers indicate next steps to improve acceptability of the referral process from pediatric ACEs screening to behavioral health.

## Introduction

Adverse Childhood Experiences (ACEs) are potentially traumatic events that occur during childhood, such as physical, emotional, and sexual abuse; neglect; and household dysfunction such as substance use and mental illness [1, 2]. It is estimated that at least 42% of the pediatric population has experienced at least one ACE [3]. ACEs and their long-term consequences were first described in a study conducted in 1998 by Kaiser Permanente and the Centers for Disease Control and Prevention (CDC) [2]. Numerous studies have shown that ACEs are associated with widespread health sequela later in life including increased health risk behaviors, developmental disruption, high risk of diabetes, heart disease, substance abuse, and overall all-cause mortality [1, 4].

Given this evidence, recent discussion of ACEs has centered around how to mitigate and prevent these effects [5]. There has been growing interest in implementing screening programs for ACEs in medical settings to identify children who are at risk, as it is suspected that potential interventions may be more effective in early childhood [6], highlighting the need for early intervention. Screening has been shown to increase identification of adversities, relative to controls, and may lead to increased referrals in those who screen positive [7].

Emerging research has investigated caregiver and patient perceptions of ACEs screening. Current literature shows that integrating ACEs screening into patient’s routine physicals was well received by patients and found to increase conversations about social influences of health [8]. Moreover, while some adolescents reported feelings of discomfort around ACEs screening, the majority of children and caregivers report that screening is comfortable and builds trust between patients and clinicians [9]. In pediatric primary care, parents were generally accepting of children being screened as a means of identifying children who could benefit from additional resources [10]. Parents also emphasized viewing pediatric primary care clinicians as an appropriate entity to perform the screening given their ability to inform parenting goals and act as change agents [10]. Some have speculated about potential harms of ACEs screening, including eroding trust between clinicians and patients, and psychological or behavioral effects of labeling children as “high risk” [11]. However, there is no data available to show if these harms have actually occurred in the setting of ACEs screening [11].

Other research focused on mitigating the harms of ACEs has focused on *intervention* when a child is at risk of adverse outcomes. Reading et al. [12] performed a qualitative review of current ACEs workflows and found that many ACEs screening workflows utilize a specific threshold for when to initiate an intervention (e.g., ACEs score and presence of ACE-related symptoms), then deploy consistent intervention strategies. For example, interventions may include counseling parents on protective factors and resiliency strategies after a child screens positive [12]. Others may include a referral for evaluation by a social worker, referral to community health workers, or referral to behavioral health professionals.

In contrast to the literature on perceptions of ACEs screenings, to date, there is little evidence on caregivers’ perspectives of ACEs interventions – in particular, ACEs-related behavioral health referrals. This information is critical to designing acceptable, caregiver- and patient-centered interventions and workflows. Thus, this study sought to better understand caregiver perceptions of the behavioral health referral process following ACEs screening for their child. Given the limited research in this area, the overarching goal was to elicit information about the facilitators and barriers facing families as they navigate receiving and following through on behavioral health referrals (i.e., obtaining behavioral health treatment for their children after a positive screen). In turn, such evidence could be used to improve the referral process and improve treatment receipt for ACE-related symptoms.

## Methods

This study was conducted at Kaiser Permanente Southern California (KPSC), a large integrated healthcare delivery system in Southern California serving more than 4.6 million members, including approximately 1 million children. Healthcare is coordinated through an integrated electronic health record (EHR) system that captures comprehensive information on the healthcare members receive at owned and contracting facilities. KPSC also obtains claims data on any out-of-network care that members receive. This study was approved by the KPSC Institutional Review Board. The study was reported in accordance with Standard for Reporting Qualitative Research (SRQR).

### Participants

Study participants were recruited from a list of children who had positive ACEs screening and were referred to behavioral health. Inclusion criteria were: any caregiver of a child referred to behavioral health due to a positive ACE score at the participating Kaiser Permanente Southern California clinic. A list of caregivers who met these criteria was generated (*N* = 151), and caregivers were called at random until a total of 20 participants was reached. Six people declined to participate, and 30 did not answer. All caregivers called were given the option to participate in the study at the time of the original call or reschedule to better fit their availability. No incentives were provided for participants. Patient confidentiality was maintained throughout this study. The list of patients who had been screened was stored on a secure server which was accessed only by the first author. Patient identifiers were not collected during the interviews, and any usage of names were extracted from the transcripts before they were uploaded to the coding software.

### ACEs screening and referral workflow

In February 2021, Kaiser Permanente Southern California (KPSC) initiated an ACEs pilot screening program at one pediatric clinic. The screening consists of a questionnaire, called the Pediatric ACEs and Related Life-Events Screener (PEARLS), completed by caregivers (for children 2–12) or the child (age 13–18) during routine pediatric appointments. The PEARLS contains questions about the 10 defined ACEs as well as questions related to discrimination, community violence exposure, food and housing insecurity, parental loss due to death, parental mental or physical disability, and separation of a parent due to foster care or immigration [13]. The PEARLS was carefully designed for sufficient patient understanding and has demonstrated adequate face validity in the pediatric primary care setting [14]. A positive screen is indicated if the child has at least one ACE and either mental or behavioral symptoms based on the clinician’s judgment. Examples of such symptoms include signs of anxiety or depression, difficulty in school, or behavioral problems, as well as persistent or untreated physical health symptoms such as asthma or obesity.

**Fig. 1:**
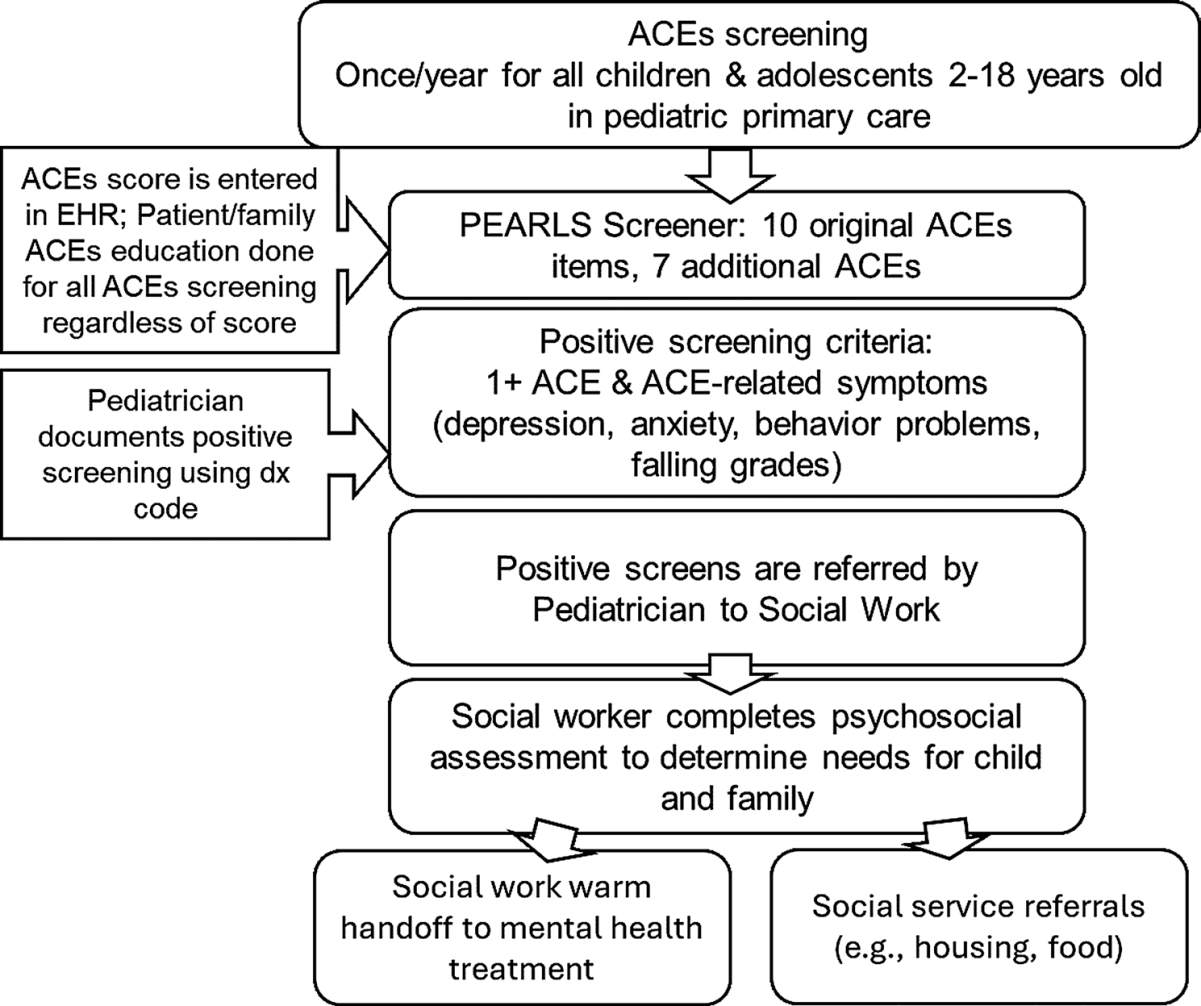
ACEs screening and referral process

The referral workflow in the clinic is as follows (Fig. 1): if a child screens positive, the clinician refers them to a pediatric social worker, who is responsible for completing a psychosocial assessment of the child and family. If indicated, the social worker provides a warm handoff, consisting of a phone call between the social worker, caregiver and behavioral health intake team, or community-based resources dependent on the individual needs of the family. Examples of such resources include food banks, parenting classes, and support groups for children with incarcerated parents.

### Research Team

A team of three researchers were responsible for designing and implementing this study. IT, a medical student, developed the standardized interview guide and conducted all interviews. MD, is a pediatrician who developed the ACEs screening program and leads the KPSC Child Abuse Prevention Program. SN is a research scientist with over 20 years of experience studying the impacts of child maltreatment and ACEs. MD and SN have developed a number of studies to test the referral pathways for ACEs screening. MD and SN provided direct oversight and input on the design and implementation of the study. All three researchers contributed to coding the transcripts and interpreting the data. None of the researchers had any established relationships with the study participants. When conducting the interviews, IT identified herself as a medical student working on improving the ACEs screening and referral process.

### Study procedure

This qualitative study was performed through phone interviews carried out from April to July 2022. While a standardized number of interviews to reach theme saturation is not agreed upon, some suggest a minimum of 20 [15]. Therefore, we conducted a total of 20 interviews before determining if data saturation had been reached.

A phenomenological approach, consisting of in-depth interviews and subsequent iterative analysis, was used to develop a semi-structured interview guide was to elicit responses of caregivers and identify key themes from the experiences of families undergoing the ACEs referral process. This approach was chosen because of its emphasis on understanding human experiences and gathering information about the specific population discussed [16]. Phenomenology typically uses open-ended questions, with the goal of gauging the true experiences of a group of people without influencing their responses. In this case, phenomenology was used to better understand the experiences of those undergoing the referral process for ACEs.

### Interview guide and conducting interviews

At the beginning of each interview, the participant provided verbal consent and was briefed about the goals of the study. Standardized interviews of approximately 25 minutes (range = 15–40 minutes) were conducted via telephone by the first author. Only the interviewer and the interviewee were present on each call. There were no repeat interviews. Further demographic information was not collected to ensure participant anonymity.

A semi-structured interview guide (Table 1) was used by the research team to query caregivers about their experience with various aspects of the ACEs screening and referral process. This guide was created using information gained from a literature search regarding general behavioral health barriers and facilitators and parent perceptions of behavioral health and therapy [17–19]. The guide was first tested in a mock interview format amongst the authors. Topics on the interview guide included caregivers’ understanding of the screening and referral process, views on behavioral health treatment, views on social worker involvement, and what challenges the families have had making or completing the referral to behavioral health. Caregivers were also asked what was helpful in the process of making or attending the appointment. Questions were ordered such that open-ended questions were asked first, followed by more specific questions about various aspects of the process. At the end of each interview, participants were asked if they had any suggestions for improving the referral process. No field notes were taken during the interviews. All interviews were audio recorded and transcribed verbatim using Microsoft TEAMS with permission from the participants. The interviewees did not review the transcripts after they were created.

**Table 1:** Summary of semi-structured interview prompts

Questions	Probing Questions
Reference: You completed a screening questionnaire about Adverse Childhood Experiences (ACEs) for your child as part of a visit with their pediatrician. -What was your understanding of the purpose of that screening? -Can you tell me what your pediatrician told you about the screening? -What did you learn about Adverse Childhood Experiences through this screening process?	
Reference: Your child was evaluated by a pediatric social worker after ACEs screening on (date), and it was determined that they could benefit from seeing a therapist or counselor or behavioral health specialist … -When you hear “mental health” or “behavioral health”, what’s the first thing that comes to mind?	Can you tell me why you feel that way? What are your feelings regarding your child receiving behavioral health treatment due to their ACE score?
What is your understanding about why your child was referred to behavioral health?	
How was the referral to behavioral health explained to you?	
How did you feel about being referred by a social worker versus a physician?	
Tell me about your experience of your child being connected to behavioral health.	What was helpful about the process of being connected to behavioral health? What made the process of being connected to behavioral health more difficult? Was there an introduction between you, the social worker, and the behavioral health specialist? Did the social worker connect you immediately? What was the length of the referral process? Was any part of the process confusing? What emotions did you feel when you were told that your child would benefit from seeing a behavioral health specialist? What could have been done to make the referral process easier for parents and their children?
If the child was never connected to behavioral health: - Why?	Did anyone ever follow up with you? What do you think would make the referral process better for parents and their children?
Has your child had a visit with behavioral health? - If not, why? - If yes, Can you tell me about your experiences with the process of making the appointment and attending the first visit?	Probes if child has not had a visit: - Were there any issues with accessing a clinician? What were they? a Were there any issues with transportation or technology that made it difficult to attend an appointment? - Were there any financial concerns regarding copays? - Did you feel supported by friends, family, or the healthcare system? Probes if child has had a visit: - What was helpful in getting you to attend the appointment? - What was difficult about attending the appointment? - Were there any issues with accessing a clinician? What were they? - How long did you have to wait for an appointment? - Was the appointment virtual or in person? - Did you feel supported by friends, family, or the healthcare system?
Has your child had more than one visit?	
Do you plan to continue visits to behavioral health?	If yes: - Do you foresee any challenges or barriers to working with behavioral health in the future? If no: - Can you tell me a bit more about why you do not want to continue working with behavioral health? - Are there any barriers to accessing behavioral health services?
Is there anything else you want to share with us that we haven’t asked?	

### Data analysis

All three authors were involved in the coding process. An initial read of the transcripts was performed by each coder prior to the coding process to familiarize themselves with the data. An inductive coding and thematic analysis method [20] was used. First, a list of codes observed in the first read of the interviews was generated. Then, a codebook was created to define and standardize the codes. As reviewed in the Introduction, a fair amount of extant literature has explored caregiver and patient perceptions of ACEs screenings. Thus, coding in the present study focused on the novel contributions – namely, perceptions of the referral process *after* ACEs screening. Dedoose software was utilized to assist with coding. A trial of coding amongst the three coders was performed with four of the transcripts to ensure adequate consensus between coders and clarify the use of each code. The focus during this process was on the agreement of the appropriate use of each code between the three coders. Any differences during this phase were resolved through discussion and revision of code definitions. Subsequently, each transcript had two randomized coders independently complete the analysis via line-by-line coding. New codes were developed and incorporated as new concepts were identified during analysis. These codes were retroactively added to previously coded transcripts as necessary. Check-ins occurred to ensure calibration to codes and discussion of any discrepancies as well as emerging themes. By the time 20 interviews were completed, saturation was reached as no new emergent codes or themes were observed in the transcripts. Interview participants did not provide feedback on the findings of this study.

After coding, code reports were generated to identify patterns emerging across transcripts. Then, the three coders met to discuss candidate themes. During these meetings, the coding patterns were discussed among the three coders in order to determine groupings across codes that may identify prominent themes. Subsequently, the candidate themes were systematically reviewed in relation to the coded data to ensure that they coalesced with the research aims, the coding, and the data. The most representative quotes for each theme are presented in Tables 2 and 3.

## Results

Of the study participants, 80% (*n* = 16) identified as the child’s parent while 20% (*n* = 4) identified as a grandparent. All caregivers that participated in the study were female. The participants ranged from 24 to 74 years old. The average age of the participants was 43.5 years old, excluding one participant who chose to not disclose their age.

The synthesized coding findings were grouped into two main categories—barriers and facilitators—under which themes were organized. Barriers were factors that made the process challenging, while facilitators were any factors that made the process easier. Under barriers, three themes were identified (Table 2): (1) Most participants described some difficulty with initial or continued contact with behavioral health, as well as challenges navigating the healthcare system, (2) There was difficulty obtaining appointment or appointment availability with the frequency desired, (3) Worry that their child would be placed on medication as part of the referral process. The first two were categorized as “structural factors” and the latter one was categorized as “individual factor.”

**Table 2:** Identified themes around barriers to ACEs referral completion and representative quotes

Barrier Theme	Number of Participants Who Mentioned Theme (*N* = 20)	Representative quotes
Structural factors		
Most participants described some difficulty with initial or continued contact with Behavioral Health, as well as challenges navigating the healthcare system	n = 16	“They tell me they’re going to call me back and then never call me.” [20]“When I do call, I have to leave a message and then I have to wait for the call back. It’s not like being able to talk to them right away … and then that’s where the phone tag comes in.” [9]
		“There’s different people I have to speak to. First the physician, then the social worker, and then the therapist. So there’s a lot of interaction with other people where, especially when you’re dealing with a situation like mine, you don’t want to have to deal with so many people.” [8]
There was difficulty obtaining appointment or appointment availability with the frequency desired	n = 11	“I think she was seeing her [therapist] once a month or once every six weeks, and that wasn’t fulfilling enough for her. So then we had to outsource.” [16]
		“It’s actually been a few months and we’re still waiting to get her in to speak to a therapist.” [9]
Individual Factors		
There was worry that their child would be placed on medication as part of the referral process	n = 4	“There are some therapists who are more likely to go towards medications and things like that. I would not want a therapist whose first option would be something of the sort. I try to avoid medications and drugs just in general for myself and for my children, so it’s not something that I would want.” [19]
		“For [Child] and as parents, we wouldn’t like her to take the medications, as the culture here in America is … for everything just medications, medications, medications.” [1]

Four themes were identified under facilitators (Table 3): (1) There were reports of feeling supported in the referral process by their healthcare team, (2) Good family and friend support was beneficial for the referral and treatment process, (3) Previous positive experience helped facilitate referral completion with behavioral health and for the caregiver to feel more comfortable with their child receiving treatment, (4) Caregivers reported that their child requesting therapy prior to the visit or the caregiver coming into the visit with a desire for a referral for their child created more open dialogue to obtain a referral. The first was categorized as a “structural factor” and the latter three were categorized as “individual factors.”

**Table 3:** Identified themes around facilitators to ACEs referral completion and representative quotes

Facilitator Theme	Number of Participants Who Mentioned Theme (*N* = 20)	Representative quotes
Structural factors		
There were reports of feeling supported in the referral process by their healthcare team	n = 6	“They’re very professional, so you don’t feel judged at all. They make you feel as if, hey, we understand this is normal and we’re here to get you to where you need to go. So I can say professionalism, compassion when speaking … and actually listening.” [16]
		“I know that they’re there and I could call at any time. I know that once I make that call, they will be able to help me in any situation.” [8]
Individual Factors		
Good family and friend support was beneficial for the referral and treatment process	n = 12	“Everybody knew that she was going into therapy. They said that it would be good for her based on what she went through earlier in her life. So they just saw the benefits for her.” [12]
		“We have a huge support system at home, and the teachers from his school and our friends that I share his journey with … Everyone is supportive of him speaking with therapists and psychiatrists.” [10]
Previous positive experience helped facilitate referral completion with Behavioral Health and for the caregiver to feel more comfortable with their child receiving treatment	n = 8	“For me, it may have been a little bit easier because I had already personally been in therapy sessions with the Kaiser therapists, so I was familiar with the process.” [16]
		“So I went through it years ago. I told [Child] that it doesn’t make you a bad person if you see a doctor like that. It’s just a different kind of doctor.” [7]
Caregivers reported that their child requesting therapy prior to the visit or the caregiver coming into the visit with a desire for a referral for their child created more open dialogue to obtain a referral	n = 10	“My daughter was having an issue with her mental health and she wanted to reach out to somebody that could help her other than her parent.” [3]
		“My kids have been through a lot of DV [domestic violence], so that’s why I asked about it … because I saw a change in their behavior.” [18]

### Barriers

Overall, the themes identified categorized under barriers centered around the difficulty with access and navigation of the healthcare system and behavioral health in particular (Table 2). There were challenges described in making initial contact as well as receiving replies to messages. In some instances, there was confusion or lack of any knowledge about who to call or the phone number to follow-up on the referral. Relatedly, there were also reports of difficulties in obtaining an appointment outside of school hours, in the modality desired (video or in-person), or with the frequency requested. Caregivers also had concerns about the financial costs of copays, travel, and missing work to take their child to appointments. This aligned with reticence to remove the child from school to attend appointments and the desire to have more availability outside school and work hours. Finally, there was concern about the uncertainty of whether their child would be placed on medication as a first option and general negative view of medication.

#### Difficulty contacting behavioral health

Most participants (*n* = 16) described some difficulty getting into, or maintaining, contact with behavioral health as well as challenges with navigating the healthcare system. There were a variety of ways in which the difficult connection to behavioral health was described by participants. Specifically, some participants reported a lack of response or miscommunication from behavioral health. For instance, there were numerous challenges described in knowing what number to call to follow-up on the referral, having their call transferred to different personnel and departments, or leaving messages without a response. There was also sentiment that having to talk to multiple people about a sensitive situation was an inconvenience and created more challenges for them. There were also discussions about being referred to clinicians external to the healthcare system and the additional challenges that arose in getting in contact with those clinicians. Moreover, some participants also experienced unexplained delays or cancellations from behavioral health. There was compounded frustration with having difficulties in making the initial appointment and then with being canceled and having to try to make the appointment again. Many caregivers described the process as being a waiting game, either in having their phone calls returned or waiting for external referrals. This difficulty with navigation seemed to be even more pronounced in cases where the child was outsourced to behavioral health outside of KPSC. Caregivers described the challenges with getting an external referral processed and then actually getting that clinician to call them back.

#### Lack of appointment availability

Over half of participants (*n* = 11) described difficulties obtaining appointments or access to appointment availability with the frequency or modality (in-person or video) desired. Some caregivers described not being able to see therapists frequently enough for the child’s needs and needing to go to an external clinician to have their child’s needs met. One caregiver noted that the external clinician had a faster turnaround to schedule an appointment. Other caregivers reported waiting long periods of time for therapists to become available for appointments. Additionally, scheduling appointments far in advance due to low appointment availability was a noted challenge with the uncertainty about the alignment with caregiver’s schedule and their future work schedule. There were noted logistical challenges related to transportation to and from appointments such as needing a behavioral health appointment in the area where the caregiver worked limited their access to appointments. There was also discussion that the child could not obtain a visit with the desired frequency, for example there were only available appointments once/month and the teenager wanted more frequency. Some caregivers also noted that they would have preferred in-person rather than a video visit, or simply just more choice in the type of visit. One child raised concerns about the loss of confidentiality with a video visit because their sibling may be home at the time. Lastly, there was repeated discussion of the need for appointments outside of work or school hours to accommodate their schedule and not have to miss work or school as well as noted concerns related to potential financial impacts of needing to miss work.

#### Medication concerns

Some caregivers (*n* = 4) mentioned feeling worried that their child would be placed on medication as part of the ACEs referral process or having concerns about the side effects. One caregiver voiced worry based on their own medication history that their child would also need it. There were also concerns that some therapists would be more likely to give medication as the first line of treatment rather than other options. There was a negative view of medications in general and the concerns that there is overmedication in the US. There was also some confusion about being told different treatment strategies, that medication would be first, then behavioral therapy, but then being told the opposite because of the child’s age.

### Facilitators

Overall, the themes identified under facilitators centered around feeling supported by family, friends, and the healthcare system enhanced the ability to feel comfortable with behavioral health treatment and follow-through with referrals. Additionally, the caregiver having previous experience with therapy or behavioral health helped in the conversations they had with their child to normalize the receipt of therapy as well as their ability to navigate the healthcare system. Finally, the child or caregiver discussion the desire for therapy prior to the visit seemed to ensure that they were in agreement and seemed to facilitate the referral process.

#### Support from the healthcare system

About one third of participants (*n* = 6) reported that they felt supported in the referral process by their healthcare team and resulted in positive feelings about the process. A few caregivers noted that the support they felt was based on having someone to answer their questions and guide them through the process. There were some healthcare system resources such as a hotline for parents to get any questions answered that was called out as particularly useful. Additionally, two caregivers specifically mentioned feeling support and understanding from healthcare workers that made them feel more normalized. They noted the professionalism and compassion shown by these healthcare workers.

#### Familial support

Most caregivers (*n* = 12) endorsed that having good support system including family, friends, and teachers helped them feel comfortable with the referral and treatment process. In some cases, caregivers explicitly mentioned this support as beneficial for the referral process. Several caregivers noted that people in their support system knew the child was going into therapy there was encouragement and reiteration of the benefits. There was also the use of friends and family as sources of informational support, with questions about what they had observed in treatment and if this was the “normal” process. Other caregivers mentioned family as positive support through stressors at home.

#### Previous positive experience with behavioral health

Almost half of caregivers (*n* = 8) mentioned positive feelings about past experiences with behavioral health or therapy. For some caregivers, the past experience was related to their own treatment. The ways in which past experience acted as a facilitator for referral completion varied. One caregiver noted that their past experience made the logistics run more smoothly. Specifically, because they had already been a patient and gone through the behavioral health intake they knew what to expect and were familiar with the process. Moreover, having that past experience allowed some caregivers to feel more comfortable having their child receive similar treatment. Caregivers discussed that their own treatment experiences encouraged them to talk to their child about it and normalize the need for mental health care. They also shared that their observations of therapy being helpful and how that facilitated their child accepting treatment.

In a few cases, the child being referred during ACEs screening had already had a past experience with behavioral health that made the family more comfortable with the referral. In a few cases their child was already seeing a therapist or had past experience with therapy. This allowed the caregiver to be open to the new referral. Lastly, two of the participants worked in the behavioral health field themselves and one caregiver noted that ACEs screening seemed less daunting due to her job and her view of the benefits of therapy.

#### Child or caregiver requesting therapy for child

Half of the caregivers (*n* = 10) reported that their child requesting therapy prior to the visit or the caregiver coming into the visit with a plan to request therapy created more open dialogue to obtain a referral. One child voiced the desire to talk with someone other than their parent. Multiple caregivers also noted that they were more responsive after hearing that the child wanted therapy. Some caregivers noted that they themselves came to the visit with the desire to obtain a referral to behavioral health due to prior or ongoing issues with their child. Conversation that occurred prior to the visit seemed to ensure that the child and caregiver were on the same page about the request for therapy seemed to facilitate positive perceptions of treatment. This agreement was viewed as beneficial for the referral process. Two caregivers also discussed how after ACEs screening, the child had brought up the idea of therapy, which prompted the caregiver to act on the referral.

## Discussion

Although emerging research suggests caregivers and patients have positive perceptions of ACEs screenings (e.g. [8–10]), little is known about how caregivers perceive interventions after screenings, such as referrals to behavioral health. This study explored caregiver perceptions of the ACEs referral process in a pediatric clinic that had implemented ACEs screenings. Although many caregivers in this study stated that the process worked well for them, there were also areas for improvement. These barriers and facilitators – as well as their implications – are described below.

Themes found in our study categorized as barriers included difficulty connecting or communicating with behavioral health, difficulty navigating the healthcare system, lack of appointment availability outside of work and school hours, and concerns that their child would be prescribed medications as the first line treatment. Notably, two themes were identified were at the system level. In other words, these barriers highlight areas where a healthcare system may be able to intervene. Examples of system-level interventions may include implementing communication workflows, providing support for people who find the multistep process to be daunting, and increasing appointment flexibility, especially outside of typical school and work hours. It is important to note that caregivers in this study identified a range of factors that made the process difficult for their family. Therefore, on a clinical level, it is important for clinicians to explore and identify individualized barriers with the families to help ensure that their concerns are addressed.

Of note, many caregivers expressed that they did not know who to contact to make appointments. In the workflow described in this study, social workers were to complete a warm handoff with behavioral health. This step was designed, in part, to allow families to easily connect with behavioral health to make appointments. Our findings suggest that there could have been instances where this step did not occur, or if that initial handoff did occur, there may be a need for continued follow up and assistance (e.g., via patient navigator programs [21]). This could alleviate some of this confusion and connection difficulty between the caregivers and behavioral health. A previous study of referrals to a family well-being clinic (FWC) after positive ACEs screening in the emergency department found that none of the families that did not receive a follow-up call initiated contact with the FWC [22]. This study, as well as our findings related to communication, emphasize that families who screen positive may need more consistent follow-up from the healthcare system. Additionally, the use of telehealth and telecommunication can potentially decrease communication-related barriers—for instance, by facilitating communication outside of appointments as well as remote monitoring [17]. However, when scaling telehealth as a tool to improve communication with patients, it is important support patients from a range of backgrounds and sociodemographics, including those who may not have access to the technology needed for telehealth.

Issues around appointment availability and access, as demonstrated as a barrier for some participants, are both well-known causes of lack of healthcare utilization. One study found that the main difference between those who complete behavioral health referrals and those who do not is financial security [19]. Additionally, numerous other studies focused on identifying barriers to behavior health referral completion have named both financial concerns and lack of appointment availability as common barriers [18, 23]. This study also demonstrated that lack of appointment *flexibility* outside of school and work hours was a hindrance to referral completion.

Additionally, a few study participants had concerns that their child would be put on medication for their mental or behavioral health challenges. While the direct cause of concern for the caregivers in this study is unknown, general concerns among caregivers about medication use in children have been well described, especially in relation to psychotropic medications [24]. Thus, when implementing workflows or interventions involving behavioral health, it is important to keep these caregivers’ concerns in mind. It is notable that medication is not a component of workflow of the participating clinic herein, yet a number of caregivers still named this concern. Clinicians may be able to address this barrier by having more in-depth conversations about the referral workflow and the role of behavioral health as incorporating multimodal strategies that incorporate a patients’ needs and preferences.

Several important facilitators related to completion of behavioral health referrals after an ACEs screening were also identified. In particular, caregivers identified familial and healthcare system support, previous positive experience with behavioral health, and the child or caregiver requesting therapy prior of ACEs screening as facilitators. Similar to the barriers, these facilitators highlight tangible ways in which healthcare clinicians can help caregivers feel supported during the multistep referral process, including being available to answer questions and providing clear instructions for contacting behavioral health. Echoing the implications from the barriers described above, these findings also suggest that referral completion rates may improve through interventions such as patient navigation programs (e.g. [21]), offering additional training to clinicians initiating ACEs screening and referral, or by creating patient handouts that are easy to read and understand. The finding that the non-judgmental attitude of healthcare workers may be a facilitator in this process is congruent with studies showing that mental health stigma can negatively impact treatment seeking behaviors and the treatment course itself [25, 26]. Additionally, one study found that help-seeking behaviors from the child, as demonstrated in our study to be a facilitator, are facilitated by past positive experiences with behavioral health and by mental health literacy [27]. Strategies to improve mental health literacy, and thereby potentially increase children’s engagement in behavioral health, may include outreach initiatives and psychoeducation, use of online multimedia campaigns, and peer training [27].

### Limitations

Limitations of this study include possible volunteer bias based on which caregivers declined to interview and which caregivers agreed to participate. We interviewed more caregivers whose children completed a visit with behavioral health than those that did not. This may have limited our understanding of other potential barriers. Moreover, the study population is a small subset of parents and caregivers in a specific health system in Southern California, and may not be fully generalizable to other locations. Because we collected limited demographic information, it is unclear whether findings capture barriers for different socioeconomic classes or traditionally marginalized groups.

## Conclusion

Improving referral completion rates after ACEs screening is still a work in progress. This study highlights the barriers and facilitators of behavioral health referral completion in children who screen positive for ACEs. Based on these study findings, possible areas of improvement include enhanced communication, providing more guidance to families through the multi-step process, increasing behavioral health specialist availability, and working with families to alleviate financial concerns. It might be helpful if clinicians participating in the referral workflow (e.g., primary care clinicians, social workers, therapists) were aware of current ACEs screening and referral workflows and their benefits/challenges in order to mitigate barriers. By addressing these barriers, and improving referral completion rates, we can potentially address the mental health symptoms related to ACEs that can have a profound impact on our patients. Important areas for future research include investigating clinician-level barriers and facilitators to referrals from ACEs screenings, assessing referral completion rates, and evaluating the patient benefits and health outcomes after completing the referred behavioral health services.

## Data Availability

Data and materials are available by reasonable request form the corresponding author and will be subject to institutional and IRB approval.
